# REal-time Assessment of Community Transmission (REACT) of SARS-CoV-2 virus: Study protocol

**DOI:** 10.12688/wellcomeopenres.16228.2

**Published:** 2021-04-21

**Authors:** Steven Riley, Christina Atchison, Deborah Ashby, Christl A. Donnelly, Wendy Barclay, Graham S. Cooke, Helen Ward, Ara Darzi, Paul Elliott, REACT study group

**Affiliations:** 1 School of Public Health, Imperial College London, London, UK; 2 MRC Centre for Global Infectious Disease Analysis, School of Public Health, Imperial College London, London, UK; 3 Patient Experience Research Centre, School of Public Health, Imperial College London, London, UK; 4 Imperial College Healthcare NHS Trust, London, UK; 5 Department of Statistics, University of Oxford, Oxford, UK; 6 Department of Infectious Disease, Imperial College London, London, UK; 7 National Institute for Health Research Imperial Biomedical Research Centre, Imperial College London, London, UK; 8 Institute of Global Health Innovation, Imperial College London, London, UK; 9 MRC Centre for Environment and Health, School of Public Health, Imperial College London, London, UK

**Keywords:** SARS-CoV-2, COVID-19, prevalence, PCR, virus, point-of-care diagnostics, lateral flow immunoassay, antibody

## Abstract

**Background:** England, UK has one of the highest rates of confirmed COVID-19 mortality globally. Until recently, testing for the SARS-CoV-2 virus focused mainly on healthcare and care home settings. As such, there is far less understanding of community transmission.

**Protocol:** The REal-time Assessment of Community Transmission (REACT) programme is a major programme of home testing for COVID-19 to track progress of the infection in the community.

REACT-1 involves cross-sectional surveys of viral detection (virological swab for RT-PCR) tests in repeated samples of 100,000 to 150,000 randomly selected individuals across England. This examines how widely the virus has spread and how many people are currently infected. The age range is 5 years and above. Individuals are sampled from the England NHS patient list.

REACT-2 is a series of five sub-studies towards establishing the seroprevalence of antibodies to SARS-CoV-2 in England as an indicator of historical infection. The main study (study 5) uses the same design and sampling approach as REACT-1 using a self-administered lateral flow immunoassay (LFIA) test for IgG antibodies in repeated samples of 100,000 to 200,000 adults aged 18 years and above. To inform study 5, studies 1-4 evaluate performance characteristics of SARS-CoV-2 LFIAs (study 1) and different aspects of feasibility, usability and application of LFIAs for home-based testing in different populations (studies 2-4).

**Ethics and dissemination: **The study has ethical approval. Results are reported using STROBE guidelines and disseminated through reports to public health bodies, presentations at scientific meetings and open access publications.

**Conclusions: **This study provides robust estimates of the prevalence of both virus (RT-PCR, REACT-1) and seroprevalence (antibody, REACT-2) in the general population in England. We also explore acceptability and usability of LFIAs for self-administered testing for SARS-CoV-2 antibody in a home-based setting, not done before at such scale in the general population.

## Background and rationale

COVID-19 is a pandemic disease caused by a novel coronavirus SARS-CoV-2. The first case in the UK was recorded at the end of January 2020 ^1^. In response to rising hospital admissions and mortality from the virus, the UK instituted a national lockdown on 23 March 2020 ^2^. Daily mortality peaked in the UK around the third week of April and then began to decline, as did the numbers of people testing positive for the virus. As of 17 August 2020, 41,369 deaths in people with a positive test had occurred in the UK ^1^.

The change from increasing to decreasing incidence was almost certainly a result of social distancing and the national lockdown ^3^. The net effect of the lockdown was to reduce the reproduction number R, the average number of new infections arising from a single infected individual, from well above 1 (estimated between 2 and 3 prior to its implementation ^4^) to below 1. As the UK transitions out of lockdown, the risk of infection in one area compared to another will be closely related to the number of infectious people in that area. As contact levels rise, an increase in R would be expected which could lead to a resurgence of infection if R again becomes greater than 1. R will vary over time and by geography. Current estimations of R are generally based on the number of cases, hospitalisation and mortality. However, sampling of transmission in the community allows a direct estimation of R.

Currently testing in the community is primarily targeted at symptomatic cases, and the uptake of this is likely to vary by geographic area, test accessibility and sociodemographic factors. Furthermore, opportunistic testing of symptomatic cases will miss asymptomatic cases who may contribute substantially to community transmission ^5^.

In addition to the need for testing of SARS-CoV-2 virus in the community, widespread testing for antibodies offers valuable monitoring of the epidemic at a population level and may provide useful insights into natural history and the sustainability of immune responses. It has also been suggested that antibody testing for individual use may assess past exposure and possibly immunity, although this is controversial until more is known about immune response ^6^. There are uncertainties around each of these uses, not least feasibility of obtaining large-scale population-wide data from representative samples, since traditional seroprevalence studies require a venous blood draw and transport of the sample to centralised laboratories, as well as assay costs.

Lateral flow immunoassays (LFIAs) offer the potential for a relatively convenient and inexpensive approach to SARS-CoV-2 antibody testing at-scale, which are easily distributed and could be self-administered at-home ^7^. There are concerns about their validity ^8^, particularly for individual use, but for population prevalence surveys it is possible to adjust for imperfect performance and obtain a reliable estimate of cumulative exposure ^9^. It is essential that, prior to use in the community, the validity of the tests is assessed, including among those who have had COVID-19 with mild or no symptoms and using finger-prick blood rather than serum. It is also important to assess their acceptability and usability as home tests. Finger-prick self-sampling has been shown to be acceptable and feasible in diabetes monitoring ^10^ and HIV testing ^11, 12^. However, the first LFIAs developed for SARS-CoV-2 antibody testing were designed as point-of-care (POC) tests undertaken by healthcare professionals. A programme to assess and maximise their acceptability and usability is required.

## Objectives and study design

The REal-time Assessment of Community Transmission (REACT) programme is designed to provide robust estimates of the prevalence of both virus (RT-PCR, REACT-1) and seroprevalence (antibody, REACT-2) in the general population in England, UK.

REACT-1 aims to provide rapid assessments of prevalence of infection in the community in England, unbiased by service factors and symptom reporting in order to assess the effects of changes to non-pharmaceutical interventions, such as easing of lockdown, on rates of infection in different areas and population groups. Repeated sampling over time allows estimates of prevalence to be obtained for different time periods which will inform estimates of R. Suitably sized samples allows national and sub-national estimates of R to detect areas with high prevalence or increasing R-values to guide the public health response.

REACT-2 aims to estimate cumulative community seroprevalence of IgG antibodies for SARS-CoV-2 in England. Antibodies provide a longer duration biomarker of exposure, relative to detection of current infection, which can help characterise the recent epidemic in more detail, including spatial and sociodemographic variation in transmission dynamics and past infection. Repeated sampling over time allows estimates of seroprevalence to be obtained for different time periods as well as changes over time, including information on antibody waning at the population-level. Large-scale antibody testing based on venous blood samples is expensive in time, personnel and laboratory resources, and cheaper more practicable approaches are required. Self-administered LFIA offers just such an approach, but this requires development work to include evaluation of performance of different LFIAs, and acceptability and usability studies of at-home self-testing among the general public and the key worker population who may be at increased risk of infection.

## Protocol

The design, sampling, sample size, outcome measures, data collection and analysis plan are described separately for REACT-1 and REACT-2, with the latter including details of five sub-studies included in the REACT-2 programme.

## REACT-1: a study of SARS-CoV-2 virus prevalence in the community in England

### Study design

Repeated cross-sectional surveys involving collection of virological swabs and reverse transcriptase polymerase chain reaction (RT-PCR) tests from a series of age-sex stratified representative population samples of 100,000 to 150,000 individuals in England. The age range is 5 years and above.

### Sampling strategy

Individuals are sampled from the NHS patient list, which includes the name, address, age and sex of everyone registered with a general practitioner in England. In order to achieve the required sample size of 100,000 or 150,000, names and demographic details of up to 750,000 individuals aged 5 years and above are randomly selected and sent personalised invitations. For children (5 to 17 years old) the invitation is sent via parents/guardians. Potential participants are invited to indicate their willingness to take part and provide informed consent either via an online portal or by telephone. Potential participants are provided with detailed information about the study including what will happen to their test and results, and how their data are managed in line with General Data Protection Regulation (GDPR) and their rights to withdraw at any point. The sample is disproportionately stratified by lower-tier local authority (LTLA) to achieve similar numbers of participants in each area.

The study includes care home residents but not individuals in detention settings.

The study samples with replacement.

### Sample size

The study is powered conservatively to give information on every LTLA in England (n=315), under the assumption that prevalence in each Local Authority is independent. There are high levels of uncertainty in populations with low prevalence. Therefore, we powered the study to provide sufficient samples in each area to inform the local administrative and public health response. With a total of 150,000 completed tests we exclude prevalence of greater than 1.2% in each area with a confidence of 95%, assuming a diagnostic sensitivity of 65% (i.e. reduced from ca. 70% in clinical settings to account for self-administration) and a diagnostic specificity of 100% ( Table 1). With 100,000 tests we exclude prevalence above 1.7% per area for the same parameters.

**Table 1.** Maximum excluded prevalence for a given number of completed tests.

Total number of tests Completed	Tests per Local Authority (n)	Maximum excluded prevalence (for 0/n)
100,000	317	1.7%
150,000	476	1.2%

### Outcomes

The primary outcome is to estimate the SARS-CoV-2 infection prevalence in the community in England. Test result (positive/negative) is used as the main outcome variable.

Secondary outcomes include:

- Quantifying geographical variation in swab-positivity rates across local authorities
- Investigating the association between swab-positivity and sociodemographic characteristics including age, gender, ethnicity and socio-economic status (SES).
- Prevalence and nature of symptoms comparing infected and uninfected individuals

### Data collection

Test kits and instructions are delivered by post to the participant’s household. Study participation involves a self-administered throat and nasal swab, and completion of a short online or telephone questionnaire including information on demographic variables, household composition, behaviour and recent symptoms. A parent or guardian takes the swab for children aged 12 years or below and also aid in questionnaire completion for children as needed. A courier arrives the same day to collect the swab, which is then sent to one of the national COVID-19 testing centres or to a commercial laboratory. Samples for the commercial laboratory use dry swabs transported on a cold chain to preserve sample integrity. Results of the RT-PCR test are sent to the participant within 24 hours of the laboratory result being received, using email, SMS and letter. Details of those with a positive test are passed to NHS Test and Trace in line with statutory requirements. If the participant is either symptomatic or has a positive RT-PCR test or both, they are instructed to self-isolate along with other members of the household according to Government advice.

### Data analysis

We calculate the prevalence of SARS-CoV-2 in each Local Authority area using two-sided binomial confidence intervals. We provide functionality in the statistical package R to produce estimates (and confidence intervals) of prevalence based on spatial aggregation of Local Authority areas, including unitary authority and regional estimates. We also provide estimates by age, sex, ethnicity and SES. Based on spatial patterns of hospital occupancy data, we expect prevalence of infection for nearby Local Authority areas to be correlated. We use a model-based geostatistical framework to investigate spatial correlation in underlying prevalence. Repeated surveys allow estimation of the changes in prevalence of infection and R-value since the easing lockdown, under assumptions of local epidemic growth rates.

To investigate association of different covariates with swab-positivity, we perform univariate logistic regression to obtain unadjusted odds ratios and 95% confidence intervals.

We also use multivariable models to adjust for age and sex and then additionally ethnicity, region, key worker status and household size.

## REACT-2: a study of SARS-CoV-2 antibody seroprevalence in the community in England

REACT-2 comprises five sub-studies to investigate feasibility, usability and application of LFIAs to measure prevalence of SARS-CoV-2 antibodies in the community. We first describe study 5, which is the measurement of IgG antibodies in random samples of the adult population in England. We then describe studies 1–4 which test different aspects of validity, feasibility, usability and application of LFIAs in different populations.

## Study 5: National seroprevalence study of SARS-CoV-2 antibodies using a lateral flow immunoassay self-administered test

### Study design

Repeated cross-sectional surveys of seroprevalence using self-administered LFIAs from a series of age-stratified representative population samples of 100,000 to 200,000 individuals in England. The age range is 18 years and above.

### Sampling strategy

Sampling is by the same method as in REACT-1.

### Inclusion criteria

Adult ≥ 18 years old.

The Medicines and Healthcare products Regulatory Agency (MHRA) did not give approval to use the lateral flow device in children in the unsupervised (at-home) setting in REACT-2. The Study Investigators and the manufacturer of the lateral flow device used in REACT-2 had to seek derogation approval for unsupervised use in adults for research purposes as it is not currently licensed for self-use.

### Exclusion criteria

Individuals with a medical condition (or are taking medication) that might increase bleeding risk from self-delivered finger prick with a lancet.

### Sample size

We aim for precision in our estimates of seroprevalence at LTLA level. Estimates are adjusted for test sensitivity and specificity of LFIAs as evaluated against clinical and laboratory references (study 1). Based on a conservative clinical sensitivity of 72% and overall population seroprevalence of 7% ^13^ we estimate the following sample sizes shown in Table 2.

**Table 2.** Lower and upper bounds 95% binomial confidence intervals (7% prevalence, sensitivity 72%).

Total number of tests completed	Lower bound (%)	Upper bound (%)
100,000	5.05	9.63
150,000	5.36	9.09

### Outcomes

The primary outcome of the study is to estimate SARS-CoV-2 IgG antibody seroprevalence in the community in England. We estimate seroprevalence as the proportion of individuals who have a positive IgG result. Test result (IgG positive/IgG negative) is used as the main outcome variable.

Secondary outcomes are as in REACT-1 with respect to antibody prevalence. In addition, the durability of immune responses is evaluated in those with proven infection.

### Data collection

Test kits and instructions are delivered by post to people who register for the study. Performing the test requires the participant to obtain a drop of blood using a lancet provided, apply this to the test cassette, add buffer provided, and read the test.

The LFIA used is chosen based on favourable performance characteristics (sensitivity and specificity) as assessed in the REACT-2 laboratory-based evaluation sub-study of LFIAs (study 1). The chosen test may change between rounds of the study dependent on improved sensitivity and specificity as more tests are trialled in the laboratory. The instructions and all public facing study material are developed with extensive public involvement and user testing (studies 2 and 3).

Participants are asked to carry out the test and follow the instructions to read the result and take a photograph of the completed test. They then complete a short online or telephone questionnaire including information on demographic variables, household composition, behaviour, recent symptoms, experience of the test and the test result. They are asked to upload the photo of the test if possible. As LFIAs are currently not approved for home testing, participants are instructed to ignore the result and continue to follow current Government advice.

Participants reporting positive antibody tests and a similar sample of those who test antibody negative and who report an invalid test result are followed up 2–4 weeks after completing the antibody test to assess COVID-19 preventive behaviour and whether these behaviours changed as a result of having read their antibody test result.

### Data analysis

Weighted estimates (and confidence intervals) of seroprevalence at LTLA, regional and national levels, together with estimates by age, sex, ethnicity and deprivation are produced. Estimates of prevalence are adjusted for known test performance using the following:

p = (q + specificity – 1) / (sensitivity + specificity – 1)

where p = adjusted proportion positive, q = observed proportion positive ^9^.

Spatial analysis and logistic regression modelling are as described for REACT-1, with respect to antibody prevalence.

A sample of participant-reported test results are checked against the photograph provided for consistency.

We perform risk factor analyses for REACT-1 and REACT-2 independently. Our list of current publications can be found on the REACT webpages: https://www.imperial.ac.uk/medicine/research-and-impact/groups/react-study/

Other analyses are planned and prioritised to deliver the most useful insights and impact on the ongoing pandemic response.

### Timelines

Study timelines to date (further rounds will depend on securing additional funding from the Department of Health and Social Care):

- REACT-1: surveys conducted monthly from May 2020 to Feb 2021 (no survey in Dec 2020)
- REACT-2 Study 5: surveys conducted Jul 2020, Aug 2020, Sep 2020, Nov 2020 and Feb 2021.

Summary of sample extraction and fieldwork dates, and response rates, by round can be found on the REACT webpages: https://www.imperial.ac.uk/media/imperial-college/institute-of-global-health-innovation/Fieldwork-info-table---REACT1-R9---REACT2-R5.pdf

Prior to the start of the COVID-19 vaccine roll out in England in December 2020, additional questions regarding vaccination status were added to the questionnaire for later rounds of REACT-1 and REACT-2. From REACT-1 Round 7 (Nov 2020) and REACT-2 Round 5 (Jan 2021) participants were being asked about COVID-19 vaccination. Study material, including the questionnaires for all study rounds can be found on the REACT webpages: https://www.imperial.ac.uk/medicine/research-and-impact/groups/react-study/

### Adverse events

There is an agreed protocol in place for reporting adverse events to the MHRA. There is a specific question asking about adverse events in the questionnaires. Participants are also provided with a study email address and contact number to use to report any adverse events.

## Study 1: Clinical and laboratory evaluation of SARS-CoV-2 lateral flow immunoassays

### Study design

Evaluation of test performance of different LFIAs.

### Sampling strategy

Clinical and non-clinical NHS employees aged 18 years or above who have previously tested positive for SARS-CoV-2 by RT-PCR are invited to take part. Participants are booked into clinic for antibody testing once they are at least 21 days from the onset of symptoms, or positive RT-PCR test, whichever is earlier.

### Inclusion criteria

1.    Adult ≥ 18 years old.

2.    Previous PCR-confirmed SARSCoV-2 (from nasopharyngeal or throat swab).

3.    Date of COVID-19 symptom onset no sooner than 21 days prior to study visit*.

*≥21 days is chosen to optimise the number of seropositives. Previous studies have shown that ELISA on sera is highly sensitive for IgG from 10 days following symptoms onset. Participants with date of positive PCR test <21 days will be asked to book an appointment (finger prick test and blood sample) for ≥21 days post PCR test.

### Exclusion criteria

Individuals with a medical condition (or are taking medication) that might increase bleeding risk from self-delivered finger prick with a lancet.

### Data collection

Five LFIAs are initially assessed, based on data from manufacturers and in the public domain in relation to sensitivity and specificity, and on availability for procurement with a view to using the best performing test in the REACT-2 national seroprevalence survey (study 5 above).

At the study appointment participants are asked to:

1. perform and interpret a self-administered LFIA under observation by a member of the research team
2. have 10 ml of venous blood drawn
3. complete a short questionnaire to include symptom history, usability and acceptability of the test

Participants are asked to interpret the results after a set time, usually 15–20 minutes as per manufacturer’s instructions, following instructions (written and pictorial) without guidance from the research team. A member of the research team records the participant’s interpretation of the test result comparing this interpretation with their own interpretation. Following self-testing, participants complete a questionnaire, including questions on sociodemographics, symptoms, date of positive RT-PCR test, and usability and acceptability of the self-test. If the participant fails any aspect of the self-test process, then the trained member of the research team performs the LFIA on the participant.

For the laboratory assessment of the five LFIAs initially assessed, and for subsequent LFIAs to be evaluated, a gold standard test for comparison of the LFIAs is made with two different laboratory-based assays:

1. laboratory tests: spike protein enzyme linked immunosorbent assay (S-ELISA) and a hybrid spike protein receptor binding domain double antigen bridging assay (Hybrid DABA);
2. a virus neutralization test that measures the biological ability of the serum to block virus infection.

A positive result in one of these serological assays is used to confirm the sample as containing SARS-CoV-2 antibody. These laboratory tests also give a readout of the quantity of antibody in each sample, allowing a determination of the cut off sensitivity of the LFIAs.

For specificity testing, 500 sera collected prior to August 2019 (negative controls) as part of the Airwave study of police personnel ^14^ are tested in each LFIA and by ELISA and virus neutralization.

### Sample size

Assuming 90% power, a COVID-19 prevalence of 100% (all participants confirmed SARS-CoV-2 positive by RT-PCR), and an expected test sensitivity of 85% we enrol a minimum of 153 participants to evaluate sensitivity with a two-sided delta of 10%.

### Outcomes

The primary outcome is the sensitivity and specificity of each LFIA. For sensitivity, tests are compared against two standards (i) RT-PCR-confirmed clinical disease (via swab testing) and (ii) positivity in patients with either a positive S-ELISA and/or positive DABA score.

LFIA performance is assessed with i) finger-prick self-testing (participant interpretation); ii) finger-prick self-testing (trained observer interpretation); and iii) serum in the laboratory. Specificity is evaluated against the known negative samples, with all positives counting as false positives.

### Data analysis

For comparison of individual LFIA performance, we compare cases where paired results from an individual are available from blood in the clinic and serum in the laboratory. We calculate sensitivities and 95% confidence intervals and test differences using the McNemar test for dependent groups. Agreement between the testing methods is assessed using the Kappa statistic ^15^.

Following selection of the LFIA for the first round of study 5, further LFIAs will continue to be evaluated for future rounds as they are developed or made available to the REACT group.

## Study 2: Public involvement and pilot testing to assess the feasibility of in-home self-testing for SARS-CoV-2 antibodies

### Study design

We use a rapid iterative participatory approach involving members of the public at all stages of the REACT programme research process.

### Sampling strategy

We recruit through existing involvement networks a small (n=20-30) but varied group of public partners to work with the research team to input into the design of the REACT-1 and REACT-2 studies. Via one or more online discussions, they advise on the development of study materials, including an instruction booklet and a short video to help people to use the LFIAs. From this network, a sub-group are sent study material via email to review and revise, including the participant information sheets, consent forms and user experience questionnaire. These advisory members are paid for their time reviewing and revising study material in accordance with NIHR INVOLVE rates ^16^.

For the LFIA usability pilot, email invitations are sent to a broader network of public contacts and volunteers who are invited to try out and feedback on the LFIA self-testing kits. This network includes a list of COVID-19 public volunteers (n=200) who have volunteered to support research and response planning at Imperial ^17^. These volunteers were recruited through VOICE-global ( https://www.voice-global.org/), an online platform for public involvement in research established by Newcastle University. Using this platform allowed us to recruit volunteers from outside of London. All public meetings were hosted online to facilitate contributions from people outside London. Furthermore, we invite participation from members of the Imperial BRC Public Advisory Panel, Imperial’s young people’s advisory group (YPAG) and members of local community organisations in North West London ^18^. Those who volunteer (up to 300) are sent a link to an online registration and consent form and asked to provide details in order for a test kit to be couriered to them with instructions. They are asked to do the antibody test and then complete an online questionnaire about their experience, similar to the questionnaire used in clinic in study 1. A subsample (n=20) are asked to perform the test while being observed via videoconferencing and take part in a short interview after completing the test.

### Inclusion criteria

Adult ≥ 18 years old.

### Exclusion criteria

Individuals with a medical condition (or are taking medication) that might increase bleeding risk from self-delivered finger prick with a lancet.

### Data analysis

Data obtained from the questionnaire on acceptability and usability are summarised by counts and descriptive statistics. Notes from the discussion groups, observations and interviews are shared within the research group and key themes and recommendations identified. The designer of the instructions/video, and the researchers responsible for kits and study materials participate in these sessions, review the results and amend the materials. Several of the public partners further review the revised materials and provide further edits.

The design and language used in the instructional video and booklet, and the decision on the type and number of lancets supplied and the type and use of pipette included in the LFIA kits for a larger population-based usability study (study 3 below) are informed by this initial public involvement and user testing phase of REACT-2.

## Study 3: Acceptability and usability study of in-home self-testing for SARS-CoV-2 antibodies in a population-based sample

### Study design

A cross-sectional study of a nationally representative sample (n=14,000) of the adult population (aged 18 years and over) in England. The study objective is to evaluate the usability of a variety of test features of LFIAs to assess whether they are suitable for home-based self-testing for SARS-CoV-2 antibodies. We evaluate two LFIAs with different usability characteristics also being validated in study 1.

### Sampling strategy

Addresses from the Postal Address File are used to draw a random sample of 30,000 households in England to which study invitation letters are sent. Based on previous work, to attain a sample of 10,000 to 15,000 people, we assumed that 1 in 3 households would opt into the study. In the invitation letter we allow up to four adults aged 18 and over in the household to register to take part in the study.

### Inclusion criteria

Adult ≥ 18 years old.

### Exclusion criteria

Individuals with a medical condition (or are taking medication) that might increase bleeding risk from self-delivered finger prick with a lancet.

### Data collection

LFIA kits are posted to each registered individual with instructions (and a link to an online video) for them to perform the test at home. On completion of the test, participants are asked to record their interpretation of the result as part of an online survey, with the option of uploading a photograph of the test result window. Further questions include information on sociodemographic characteristics and questions around the acceptability and usability of the kits.

### Outcomes

The main study outcome is usability of the LFIA kits. This is evaluated according to participants’ ability to understand the instructions, use the kit to obtain a valid test result, and to correctly interpret the result in a home-based setting.

To determine how accurately participants interpret their test result, we look at the agreement between a participant’s self-reported test result compared with a clinician’s interpretation of the same result. The clinician, blinded to the participant’s interpretation, examines the photograph submitted for all results reported positive or unable to read, and a random sample of 200 within each of the negative and invalid test outcome categories.

Acceptability is defined as consenting to and using the provided self-test in the participants’ homes. There is also a further question asking participants whether they would be willing to repeat a self-administered finger-prick antibody test in the future. Other secondary outcomes include whether participants had assistance to do the test, reasons for not completing the test, participants’ preferences for home-based self-testing versus clinic or community centre-based testing and whether parents would be willing to carry out the test on their children.

### Data analysis

Data obtained from the questionnaire on acceptability and usability are summarised by counts and descriptive statistics. Agreement between participant-interpreted and clinician-interpreted result is assessed using the Kappa statistic ^15^.

The decision to proceed with an LFIA, and which one, for the large-scale national seroprevalence study (study 5) will be based on a number of criteria including the usability and acceptability determined in this study, a relatively low proportion of invalid results, a high concordance with clinician-read results, together with test performance in the laboratory evaluation of clinical samples (study 1).

## Study 4: Usability and validity of LFIA self-testing in key workers, including the assessment of dry blood spots for SARS-CoV-2 antibody detection and saliva for SARS-CoV-2 viral detection

### Study design

A cross-sectional study to assess accuracy, acceptability, usability and feasibility of self- and healthcare professional administered LFIA among key workers. In addition, this study assesses the use of dry blood spots as an alternative means of testing antibody response and use of a saliva sample compared with a throat and nose swab for RT-PCR testing.

### Sampling strategy

Key workers are invited to participate: up to 5,500 individuals aged 18 years and above. These key workers are police officers and staff (Airwave Study participants ^14^), and members of the fire service.

Potential participants from the study population are sent an invitation letter and a participant information sheet by post or email. They are asked to contact the research team to make a clinic appointment at one of the six study sites (London, Manchester Warwick, Derby, Keele and Bournemouth) and followed up with one reminder letter or email.

### Inclusion criteria

Adult ≥ 18 years old.

### Exclusion criteria

Individuals with a medical condition (or are taking medication) that might increase bleeding risk from self-delivered finger prick with a lancet.

### Data collection

The participant is invited to conduct the LFIA test themselves in clinic without direct instruction from a staff member. The participant is asked to provide a finger-prick blood sample (1 drop) and follow the kit instructions provided, including photographing the result and uploading the photograph via a secure web portal. In addition, the participant is asked questions concerning usability and acceptability of the device as per other sub-studies in REACT-2. Participants are then asked to repeat the test, administered by a trained healthcare professional. A photograph of the test result is taken and uploaded via the secure web portal.

The healthcare professional also collects from participants:

1. Up to 5 spots of blood from the participant’s finger that is lanced onto a dry blood spot (DBS) storage card. The blood spot card is allowed to dry for a minimum of four hours before it is shipped to the laboratory for storage in a humidity-controlled environment.
2. Venous blood samples (40 ml from existing members of the Airwave Study / 10 ml from all other study participants). This blood is a source of serum and plasma that is used for validation of SARS-CoV-2 antibody testing technologies. The additional blood collected from Airwave Study participants is used for haematology, clinical chemistry (e.g. serum cholesterol and HbA1C) and storage.

Participants are also asked to provide for RT-PCR testing:

- 2 ml of saliva into a pot.
- A self-swab of their throat and nose.

A video is played to the participants at the centre, providing clear instructions on the swabbing procedure. The self-swabbing is done in a private car before or after their appointment (as convenient) or under a shelter with two open sides (as used in drive up testing hubs). The participant is provided with a sealed bag and asked to drop their saliva pot and swab into a non-contact locked clinical waste bin. The laboratory technician wearing appropriate personal protective equipment (PPE) transfers the samples to cooled shipping containers at regular intervals. The saliva and swab are shipped to analytical laboratories each day.

The results of the throat and nose swab RT-PCR test and the laboratory antibody test are sent to participants. Participants are clearly advised that the results of both the LFIA and laboratory antibody test are for research purposes only. They are instructed to continue to follow UK government advice. The results of the RT-PCR analysis of the nose and throat swab indicate if the participant currently has an active SARS-CoV-2 infection or not. If the test result is positive, it is communicated to the participant via an email, text or phone call, within 3–4 days of their appointment. In practice, PCR results are communicated within 48 hours to participants. We specify “within 3–4 days” to manage expectations and potential laboratory processing delays due to increased demands on testing during the pandemic.

Details of those with a positive test are passed to NHS Test and Trace in line with statutory requirements. If the participant is either symptomatic or has a positive RT-PCR test or both, they are instructed to self-isolate along with other members of the household according to Government advice.

### Sample size

We consider statistical power for the LFIA and possible heterogeneity by job type, age and sex. For 80% power and 95% confidence to detect differences between the sensitivity of the antibody tests with the healthcare professional-administered test at 65% and the participant-administered test at 55%, we require 222 individuals per healthcare professional/participant-led arms, giving 20 classes (2 genders, 10 age groups, and officers/staff) and a minimum of 4,440 participants. Additional participant numbers allow for test failures and differences in sensitivity from those estimated above. For the RT-PCR testing at 0.5% prevalence 5,500 participants gives around 27 positive cases.

### Outcomes

LFIA performance, acceptability and usability among key workers are assessed with i) finger-prick self-testing; ii) finger-prick healthcare professional-administered testing; and iii) plasma in the laboratory.

These data provide evidence as to whether LFIAs should be used as a self-administered test among key workers or whether better performance is obtained if they attend a central clinic facility, e.g. for blood draw.

### Data analysis

For comparison of individual LFIA performance, we compare cases where paired results from an individual are available from the clinic and laboratory. We calculate sensitivities and 95% confidence intervals and test differences using the McNemar test for dependent groups. Agreement between the testing methods is assessed using the Kappa statistic ^15^.

Comparison of results from the throat and nose swab and saliva RT-PCR test is used to investigate the use of saliva as an alternative means of obtaining a virological sample that could be safely collected in the home setting.

We examine prevalence of RT-PCR and antibody positive test by age, sex and job type, comparing participant versus healthcare professional administration of the LFIA test. In addition, antibody results from plasma are used to compare with those from the use of the dry blood spot which could be self-administered at home as an alternative means to detecting SARS-CoV-2 antibodies.

## Ethics, consent and public involvement

The REACT programme studies obtained research ethics approval from the South Central-Berkshire B Research Ethics Committee (IRAS ID: 283787). Participants provide informed consent when they register for the studies, and all data are handled securely in accordance with a detailed privacy statement. A REACT Study Public Advisory Panel meets fortnightly to provide ongoing input into the research design, delivery and dissemination.

## Plans for dissemination

To ensure that the outputs from the research inform and support the international, national and local public health response to COVID-19 in a timely manner, we will publish preprints of the main study findings. Reports are submitted weekly to key stakeholders, including the UK Department of Health and Social Care, and are fed into key government committees. We also work with our Public Advisory Panel to identify and produce materials, including infographics and blogs, to disseminate our findings to non-academic audiences and the general public.

## Conclusions

The REACT programme is a series of studies that are seeking to improve understanding of how the COVID-19 pandemic is progressing across England. To do this, the programme is carrying out two major pieces of work that are looking at the possibility of using home sampling and testing to track the infection. REACT-1 examines, over time, how many people across England are currently infected with SARS-CoV-2. REACT-2, firstly assesses a number of different antibody tests to see how accurate they are and how easily people can use them at home. Then, a large national seroprevalence study is rolled out to explore how far the virus has spread across the country and what proportion of the population have been infected and recovered.

Together REACT-1 and REACT-2 will improve our understanding of the transmission of the virus in the community to help guide policies on continued social distancing and other control measures.

## Data availability

No data are associated with this article.
