# Supplementary material for: REal-time Assessment of Community Transmission (REACT) of SARS-CoV-2 virus: Study protocol
Source: Wellcome Open Res. 2021 Apr 21;5:200. Originally published 2020 Aug 25. [Version 2] doi: 10.12688/wellcomeopenres.16228.2 (PMC8095190; doi:10.12688/wellcomeopenres.16228.2)
Supplement: Peer review history

# Peer review: Reviewer response for version 2

**DOI:** 10.21956/wellcomeopenres.18530.r43557

**Published:** 2021-04-30

**Authors:** Dan Hungerford

**Affiliations:** 1 Institute of Infection and Global Health, University of Liverpool, Liverpool, UK

The authors have provided detailed responses to my questions and updated the protocol appropriately.

Is the study design appropriate for the research question?

Yes

Is the rationale for, and objectives of, the study clearly described?

Yes

Are sufficient details of the methods provided to allow replication by others?

Yes

Are the datasets clearly presented in a useable and accessible format?

Not applicable

Reviewer Expertise:

Infectious disease epidemiology.

I confirm that I have read this submission and believe that I have an appropriate level of expertise to confirm that it is of an acceptable scientific standard.

# Peer review: Reviewer response for version 1

**DOI:** 10.21956/wellcomeopenres.17824.r41593

**Published:** 2020-12-14

**Authors:** Dan Hungerford

**Affiliations:** 1 Institute of Infection and Global Health, University of Liverpool, Liverpool, UK

This study protocol covers a programme of studies in England to assess SARS-CoV-2 infection rates in the community over time through large cross-sectional sampling. Using the same sampling methodology it also intends to assess seroprevalence of antibodies to SARS-CoV-2 in the community in England as an indicator of prior exposure to SARS-CoV-2 infection and assess how levels of exposure change in England over time. The study is using self-administered tests, nasal and throat swabs for viral RT-PCR detection and lateral flow immunoassay for IgG antibodies.

 This is clearly a challenging and impressive piece of work that has been planned, that adds value to a number of areas of understanding of COVID-19 disease transmission and SARS-CoV-2 infections in the community. It will also provide critical information on understanding how members of the public interact with self-administered home tests for infectious diseases, and therefore will add value outside of the SARS-CoV-2 pandemic as well.

 The protocol is well written and includes enough detail to follow the procedures. The aims of study are clearly laid out. It already has ethical approval and has PPI, which reassures me that it has already been through rigorous review both internally and externally. Therefore the comments I have made below are minor and may just require extra clarification either in response to my comments or in the protocol.

- How does the study intend to deal with vaccine recipients or those enrolled on vaccine trials? This should be included in the protocol.
- Please provide the study timelines and frequency of sampling for both REACT-1 and REACT-2. Also, will the study sample with replacement?
- Will the studies exclude participants from institutional settings such as care homes/prisons?
- How will the study deal with adverse advents related to testing in participants?

**REACT-1**

- Pg 4 Sample size – It is a large assumption to assume independence within each LA particularly in metropolitan areas and tourist destinations can the authors justify this assumption?
- Pg4 Secondary outcomes – Household composition/size, occupation, schooling and behaviours (out of house contact), seem to not be included, is this intentional as these would seem like key variables?
- How will they manage clinical illness or swab positive illness in complicated scenarios (e.g. key workers, those working with vulnerable groups), will this be left to test and trace? Will a clinician be available to advise positive participants?
- Pg 4 Data analysis – How will multivariable models be specified particularly secondary outcomes, will you use *apriori* decisions, stepwise approaches etc.?

**REACT-2 Study 5**

- My main question is why are children excluded from the study? This seems a shame to miss such an important part of the population?
- Outcomes – Would it not be of interest to come risk factor analysis in seropositive compared to swab positive populations?
- Are any questions asked about whether participants believe they have previously had SARS-CoV-2 or they have had a positive test previously?

**REACT-2 Study 2**

- The volunteers seems to be very London centric, would it not be sensible to open it open to groups outside of London, as this is meant to be a England wide study?

**REACT-2 Study 4**

- Pg 8 - It says that study results will be communicated within 3-4 days, why the delay? This is much slower than in REACT-1 and in current pillar 2 testing. Since these are key workers a rapid result would be far preferable should public health action need to be taken.

Is the study design appropriate for the research question?

Yes

Is the rationale for, and objectives of, the study clearly described?

Yes

Are sufficient details of the methods provided to allow replication by others?

Yes

Are the datasets clearly presented in a useable and accessible format?

Not applicable

Reviewer Expertise:

Infectious disease epidemiology

I confirm that I have read this submission and believe that I have an appropriate level of expertise to confirm that it is of an acceptable scientific standard, however I have significant reservations, as outlined above.

# Response

**Published:** 2021-03-31

**Authors:** Christina Atchison

**Affiliations:** School of Public Health, Imperial College London, UK

**Response to reviewer comments (General)**

**How does the study intend to deal with vaccine recipients or those enrolled on vaccine trials? This should be included in the protocol.**

 Prior to the start of the vaccine roll out in England in December, additional questions regarding vaccination status were added to the questionnaire for later rounds of REACT-1 and REACT-2. From REACT-1 Round 7 (Nov 2020) and REACT-2 Round 5 (Jan 2021) participants were being asked about coronavirus vaccination. Study material, including the questionnaires for all study rounds can be found on the REACT webpages: https://www.imperial.ac.uk/medicine/research-and-impact/groups/react-study/

**Please provide the study timelines and frequency of sampling for both REACT-1 and REACT-2. Also, will the study sample with replacement?**

 The study samples with replacement.

 Study timelines to date (further rounds will depend on securing additional funding from the Department of Health and Social Care):

 REACT-1: surveys conducted monthly from May 2020 to Feb 2021 (no survey in Dec 2020)

 REACT-2: surveys conducted Jul 2020, Aug 2020, Sep 2020, Nov 2020 and Feb 2021.

 Summary of sample extraction and fieldwork dates, and response rates, by round can be found on the REACT webpages: https://www.imperial.ac.uk/media/imperial-college/institute-of-global-health-innovation/Fieldwork-info-table---REACT1-R9---REACT2-R5.pdf

**Will the studies exclude participants from institutional settings such as care homes/prisons?**

 The studies include care home residents but not individuals in detention settings.

**How will the study deal with adverse advents related to testing in participants?**

 There is an agreed protocol in place for reporting adverse events to the Medicines and Healthcare products Regulatory Agency (MHRA). There is a specific question asking about adverse events in the questionnaires. Participants are also provided with a study email address and contact number to use to report any adverse events.

**Response to reviewer comments (REACT-1)**

**Pg 4 Sample size – It is a large assumption to assume independence within each LA particularly in metropolitan areas and tourist destinations can the authors justify this assumption?**

 We didn’t know what the spatial correlation structure would be in these data, nor that it would even be constant. Therefore, we thought that direct use of the data for public health would start from independent estimates of local prevalence, hence the power calculation was made to ensure that the sample was large enough that there would be useful data in each local area.

**Pg4 Secondary outcomes – Household composition/size, occupation, schooling and behaviours (out of house contact), seem to not be included, is this intentional as these would seem like key variables?**

 These were included in the questionnaire. Study material, including the questionnaires for all study rounds can be found on the REACT webpages: https://www.imperial.ac.uk/medicine/research-and-impact/groups/react-study/

**How will they manage clinical illness or swab positive illness in complicated scenarios (e.g. key workers, those working with vulnerable groups), will this be left to test and trace? Will a clinician be available to advise positive participants?**

 The study formally reports all positives to Public Health England to be passed on to NHS Test and Trace in exactly the same way as positives arising from Pillar 2 testing. This is explained to participants during the consent process.

**Pg 4 Data analysis – How will multivariable models be specified particularly secondary outcomes, will you use apriori decisions, stepwise approaches etc.?**

 We use a core set of variables for the multivariable model and then consider additional variables when prevalence is high enough. The core variables are: sex, age, region, ethnicity, work type, and household size.

**Response to reviewer comments (REACT-2)**

**REACT-2 Study 5**

**My main question is why are children excluded from the study? This seems a shame to miss such an important part of the population?**

 MHRA did not give approval to use the lateral flow device in children in the unsupervised (at-home) setting in REACT-2. The Study Investigators and the manufacturer of the lateral flow device used in REACT-2 had to seek derogation approval for unsupervised use in adults for research purposes as it is not currently licensed for self-use.

**Outcomes – Would it not be of interest to come risk factor analysis in seropositive compared to swab positive populations?**

 We have performed risk factor analyses for REACT-1 and REACT-2 independently. Our list of current publications can be found on the REACT webpages: https://www.imperial.ac.uk/medicine/research-and-impact/groups/react-study/

 Other analyses are planned and prioritised to deliver the most useful insights and impact on the ongoing pandemic response.

**Are any questions asked about whether participants believe they have previously had SARS-CoV-2 or they have had a positive test previously?**

 Yes, we ask questions regarding previous SARS-CoV-2 infection. Study material, including the questionnaires for all study rounds can be found on the REACT webpages: https://www.imperial.ac.uk/medicine/research-and-impact/groups/react-study/

**REACT-2 Study 2** ****

**The volunteers seems to be very London centric, would it not be sensible to open it open to groups outside of London, as this is meant to be a England wide study?**

 Volunteers were recruited through existing public involvement networks, including a list of COVID public volunteers (n=200) who volunteered to support research and response planning at Imperial. These volunteers were recruited through VOICE-global ( https://www.voice-global.org/), an online platform for public involvement in research established by Newcastle University. Using this platform allowed us to recruit volunteers from outside of London. All public meetings were hosted online to facilitate contributions from people outside London.

**REACT-2 Study 4**

**Pg 8 - It says that study results will be communicated within 3-4 days, why the delay? This is much slower than in REACT-1 and in current pillar 2 testing. Since these are key workers a rapid result would be far preferable should public health action need to be taken.**

 In practice study PCR swab results were all communicated within 48 hours to participants. We specified “within 3-4 days” in the protocol to manage expectations and potential delays if the laboratory ran into capacity issues during the pandemic with increased demands on testing.

# Peer review: Reviewer response for version 1

**DOI:** 10.21956/wellcomeopenres.17824.r40921

**Published:** 2020-11-09

**Authors:** Julie Parsonnet

**Affiliations:** 1 Division of Infectious Diseases, Department of Medicine, Stanford University School of Medicine, Stanford, CA, USA

I've already seen some of the results of this work posted on a preprint server - it is important to get the methods out and published so that the study design can be referenced and understood as results become available.

 This is a clearly written paper about an important epidemiological study. I think the study may have some problems down the line, particularly with participation of certain population groups. However, that is a problem inherent in many "population-based" research studies that rely on willingness of people to join the research. 

 The last epidemiological data for this study are from August 17, 2020. It would be nice to see this updated.

 What is the magnitude of change in RT-PCR prevalence or seroprevalence or that will be detectable in these studies? Are the sample sizes sufficient to assess population subgroups by age or ethnicity?

 Why do the investigators use 2019 controls for their testing of the LFIA's specificity rather than asking negative subjects to perform the tests? What if they can't read a negative? Also, LFIA's when done wrong, usually come out negative. For example, if no blood is put in the well, many LFIA's would read that as a negative result (not an inadequate test). If that is the case, whether an observer reads the test or the subject, the results will be wrong either way. They may be concordant, but they will be wrong. How do the investigators assess this problem?

 It would be good to see the previous work showing 1/3 would participate. This is far more than what is being seen in US studies.

 Also, by only testing sensitivity of serologies 21 days after RT PCR diagnosis, the authors are maximizing test sensitivity. In reality, people at home will, not infrequently as rates rise, be in the first week or two of infection. How do the investigators expect to control for this possibility? Also, serology reflects a survival bias, particularly among the elderly.

 Overall, I'm very enthusiastic to see this paper indexed. The paper is sound as is and these are minor questions.

Is the study design appropriate for the research question?

Yes

Is the rationale for, and objectives of, the study clearly described?

Yes

Are sufficient details of the methods provided to allow replication by others?

Yes

Are the datasets clearly presented in a useable and accessible format?

Not applicable

Reviewer Expertise:

Infectious diseases epidemiology.

I confirm that I have read this submission and believe that I have an appropriate level of expertise to confirm that it is of an acceptable scientific standard.
